# A Predictive Strength Model for Cu/Ni Nanolayered Composites with FCC Interfacial Layers Under Loading Parallel to the Interface

**DOI:** 10.3390/nano16171106

**Published:** 2026-09-02

**Authors:** Yaodong Wang, Jianjun Li

**Affiliations:** 1 State Key Laboratory of Intelligent Construction and Healthy Operation and Maintenance of Deep Underground Engineering, School of Mechanics and Civil Engineering, China University of Mining and Technology, Xuzhou 221116, China; 2 School of Mechanics and Engineering Science, Ningbo University, Ningbo 315211, China

**Keywords:** nanolayered composites, strength, interfacial layers, molecular dynamics

## Abstract

Nanolayered metallic composites exhibit ultra-high strength but suffer from inadequate ductility. Constructing interfacial layers becomes an effective strategy to enhance their strength and ductility. However, extensive experimental studies have focused on the strengthening and toughening mechanism of some special interfacial layers under loading normal to the interface, a systematic understanding of how interfacial layer characteristics influence the mechanical response remains unclear, especially under loading parallel to the interfaces. Here, molecular dynamics simulations using the LAMMPS code with embedded atom method potentials are performed to investigate the tensile deformation of Cu/Ni nanolayered composites with various interfacial layers made by six different FCC metals, i.e., Ag, Al, Au, Pb, Pd, and Pt, under loading parallel to the interfaces. Our simulation results reveal that the strength of composites is governed by the metallic element of interfacial layers. The composites with Pd and Pt interfacial layers exhibit the highest and lowest strength, respectively, showing a maximum strength difference of 1.09 GPa. The strength variation is attributed to the synergistic interplay of multiple characteristics of interfacial layers, rather than from a single dominant factor. Furthermore, a quantitative mapping relationship between the strength of composites and the characteristic parameters of the interfacial layers was established on the basis of the Voigt model and the dislocation nucleation behavior. Accordingly, the strength can be expressed as a function of three decisive determinants: the strain energy density required for dislocation nucleation in the pure metal corresponding to the interfacial layers, the elastic modulus of that pure metal, and a parameter related to the stress concentration level at the interfaces. A higher value of the former two factors, combined with a lower value of the latter, corresponds to a higher strength.

## 1. Introduction

Nanolayered metallic composites exhibit exceptional properties, including ultra-high strength [1,2,3], high electrical conductivity [4,5,6], excellent radiation damage tolerance [7,8,9], and good thermal stability [10,11,12]. These advantages make them promising candidates for applications in micro-electro-mechanical systems (MEMS) [13,14], surface-enhanced Raman scattering substrate materials [15], and the nuclear industry [16,17]. Beyond these inherent merits, their mechanical performance can be further enhanced through microstructural design, such as the introduction of nanotwins [18,19] and the construction of heterogeneous structures [20,21,22]. Modulating the strength and ductility of nanolayered metallic composites by precisely constructing microstructures has become a hot research topic [23,24,25,26].

In recent years, the incorporation of interfacial layers into nanostructured metallic materials has emerged as an effective strategy for improving strength and ductility [27,28,29]. This approach has been extended to nanolayered metallic composites in the form of interfacial layers and three-dimensional (3D) interfaces [30,31,32]. For instance, Chen et al. [33] prepared Cu/Nb nanolayered composites with 3D CuNb interfaces by physical vapor deposition. The 3D interfaces were fabricated by linearly modulating the sputtering power of Cu and Nb targets during co-sputtering. Micropillar compression tests revealed that the samples with 3D interfaces exhibited a 50% higher yield strength than their 2D counterparts. Using a similar deposition approach, Leo et al. [34] prepared Ti/Nb nanolayered composites with 3D interfaces. The 3D sample reaches a hardness of 5.79 GPa, which is higher than that of the 2D samples (4.5 GPa). Similarly, Cheng et al. [35] prepared Cu/Nb nanolayered composites with 3D interfaces. They found that the 3D samples show enhanced strength and deformability compared with 2D samples, under compression applied perpendicular to the interfaces and at a 45° angle to the interfaces. In addition to strength and ductility, the addition of interfacial layers can also enhance thermal stability [36,37,38]. For instance, Kim et al. [39] fabricated Ni/Ti nanolayered composites with graded 3D interfaces using direct current magnetron sputtering. They demonstrated that these 3D interfaces exhibit superior microstructural stability, even after annealing at 500 °C for 1 h, and act as a kinetic buffer against interdiffusion-induced degradation.

To probe the underlying mechanisms, Fuchs-Lynch et al. [40] developed a phase-field dislocation dynamics (PFDD) model and applied this model to simulate the slip transfer of dislocations across both sharp 2D and thick 3D bi-phase interfaces in Ti/Nb nanolayered composites. Their results demonstrated that higher critical stress is required for transfer across the 3D interfaces than for the 2D interfaces. Wang et al. [41] investigated the tensile deformation of Cu/Ni nanolayered composites with Ag interfacial layers by molecular dynamics simulations. They found that the addition of interfacial layers alleviates the stress concentration at the interfaces, which strengthens materials by rendering dislocation nucleation more difficult. Dong et al. [42] studied the deformation behavior of Cu/Nb nanolayered composites with 3D interfaces by using MD simulations. They revealed that pre-existing dislocation sources in the 3D interfaces activate more dislocation slip planes, thereby effectively suppressing shear localization.

Existing studies have demonstrated that a variety of interfacial layers can enhance the strength of nanolayered composites. However, it remains unclear which structural or physical characteristics of the interfacial layers dominate the strengthening effect. Here, the tensile deformation of Cu/Ni nanolayered composites with interfacial layers made by typical FCC metals (Ag, Al, Au, Pb, Pd, and Pt) under loading parallel to the interfaces is investigated by molecular dynamics simulations. Our simulation results reveal that the strength of composites varies with the metallic elements of interfacial layers, following a descending order of Pd, Ag, Au, Pb, Al, and Pt. Further analysis indicates that the strength variation across different interfacial layers originates from the combined variation in elastic modulus and elastic strain. The elastic modulus of the composites increases linearly with that of the interfacial metals, in good agreement with the Voigt model. Based on the energy evolution during dislocation nucleation, a quantitative relationship between the elastic strain and the interfacial characteristic parameters is established, in which the elastic modulus is codetermined by the strain energy density required for dislocation nucleation of the pure metal corresponding to the interfacial layers, the elastic modulus of that pure metal, and a parameter associated with stress concentration level at interfaces.

## 2. Simulation Methodology and Model

**Figure 1:**
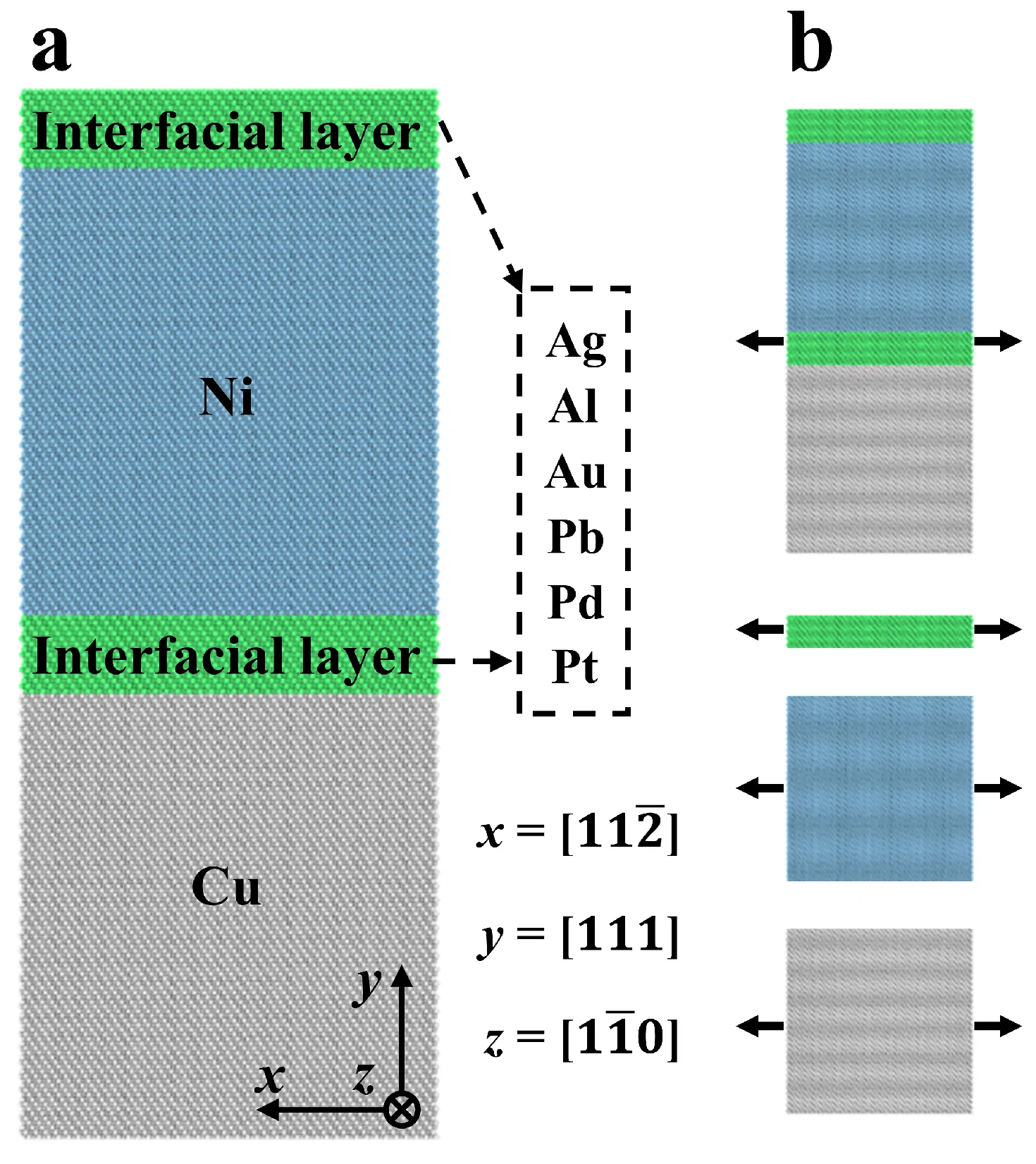
Schematic illustration of the simulation model. (**a**) Configuration of Cu/Ni nanolayered composites with different interfacial layers. (**b**) Tensile simulation of the composites and their constituent layers under loading parallel to the interface.

The tensile deformation of Cu/Ni nanolayered composites with six different FCC interfacial layers under the loading parallel to the interfaces was simulated by molecular dynamics. Figure 1a shows the configuration of simulated samples, where Cu layer, interfacial layers, Ni layers, and interfacial layers were stacked sequentially. Six interfacial layers were made by Ag, Al, Au, Pb, Pd, and Pt, respectively, and the corresponding samples were denoted as Cu/Ni-Ag, Cu/Ni-Al, Cu/Ni-Au, Cu/Ni-Pb, Cu/Ni-Pd, and Cu/Ni-Pt, respectively. All metals possessed face-centered cubic (FCC) structures and shared the same crystallographic orientation, namely $x=[11\bar{2}]$, y=[111], and $z=[1\bar{1}0]$. The atomic radii (*d*) of Cu, Ni, Ag, Al, Au, Pb, Pd and Pt were 0.3615 nm, 0.35196 nm, 0.40896 nm, 0.40502 nm, 0.40801 nm, 0.49494 nm, 0.38904 nm, and 0.39201 nm, respectively, which were obtained from the potentials used in our simulations [43]. The dimensions of minimum unit cells along the *x*-, *y*-, and *z*-directions are $\frac{\sqrt{6}}{2}d$, $\sqrt{3}d$, and $\frac{\sqrt{2}}{2}d$, respectively. Layers are obtained by duplicating the minimum unit cell, resulting in a size mismatch along *x*- and *z*-directions between layers in a single sample due to the different *d*. The final dimensions along *x*- and *z*-directions are selected to minimize the size mismatch, ensuring that no defects are observed at the periodic boundary after energy minimization and relaxation. The layer thicknesses of the Cu, Ni, Ag, Al, Au, Pb, Pd, and Pt layers were 20.04 nm, 20.11 nm, 3.54 nm, 3.51 nm, 3.53 nm, 3.43 nm, 3.37 nm, and 3.39 nm, respectively. These simulated samples were constructed using the ATOMSK package [44]. The final dimensions of the simulation samples were 18.6 nm×47.2 nm×19.7 nm for the Cu/Ni-Ag sample, 16.8 nm×47.2 nm×18.9 nm for the Cu/Ni-Al, 15.5 nm×47.2 nm×18.1 nm for the Cu/Ni-Au, 16.3 nm×47 nm×18.9 nm for the Cu/Ni-Pb, 18.2 nm×46.9 nm×18.1 nm for the Cu/Ni-Pd, and 16.8 nm×46.9 nm×19.4 nm for the Cu/Ni-Pt samples.

As illustrated in Figure 1b, the loading was applied along the *x*-direction, which are parallel to the interfaces. All tensile simulations were performed using the Large-scale Atomic/Molecular Massively Parallel Simulator (LAMMPS) [45]. The Embedded Atom Method (EAM) potential developed by Zhou et al. [43] was employed to describe the interatomic interactions, which yields results in good agreement with experimental data [46,47]. The simulations were run with a time step of 1 fs. Periodic boundary conditions were applied in all directions. Each sample was first energy-minimized and equilibrated in an NPT ensemble at 50 K and then subjected to uniaxial tension at a constant strain rate of $5\;\times \;10^{8}{\;s}^{-1}$ up to a total strain of 15%. The system temperature and pressure were controlled by the Nose–Hoover thermostat and barostat, respectively. Simulations of constituent layers of the composites were also performed for comparison. Atomic information was visualized and analyzed by using the OVITO software package (v3.15.4) [48].

## 3. Results and Discussion

**Figure 2:**
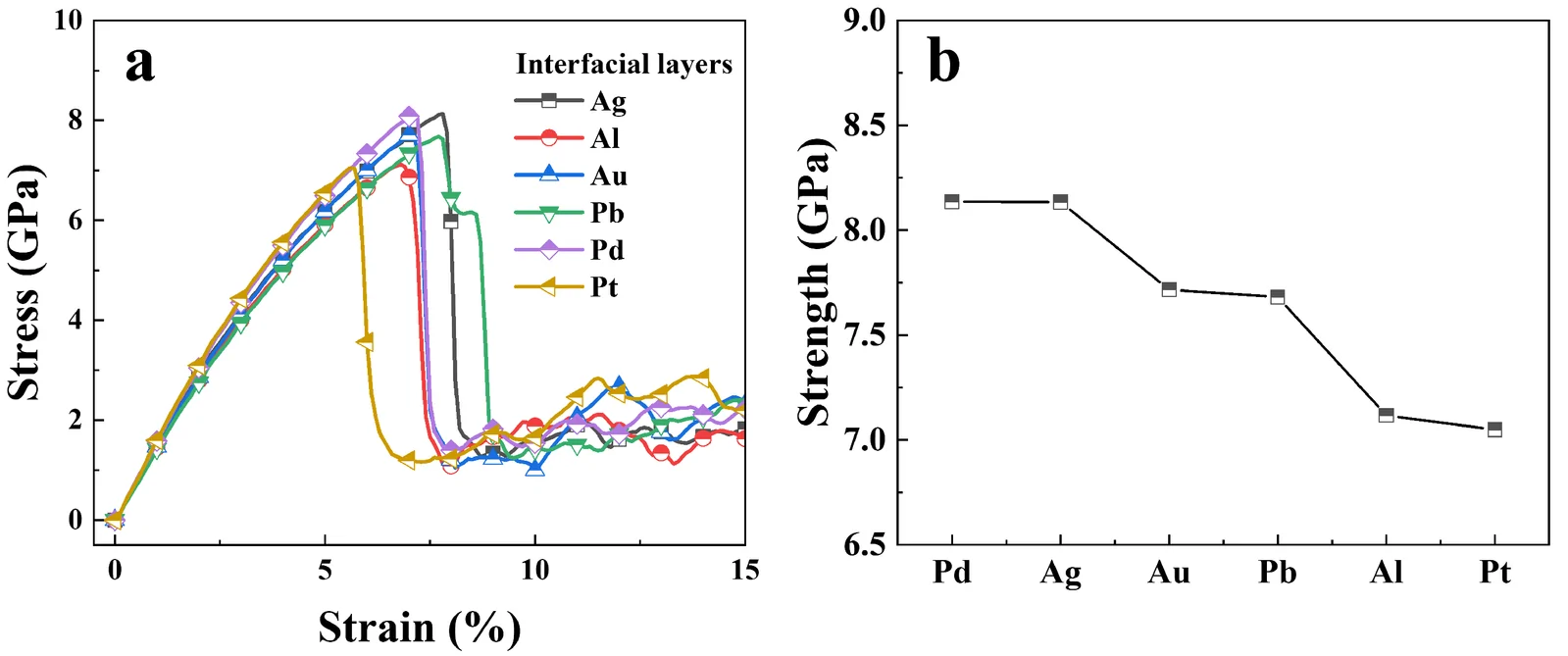
Tensile response of Cu/Ni nanolayered composites with different interfacial layers. (**a**) Stress–strain curves. (**b**) Variation in strength with different interfacial layers.

Figure 2a shows the stress–strain responses of Cu/Ni nanolayered composites with different interfacial layers under tensile loading parallel to the interface. Stress increases with increasing strain until reaching a peak value, followed by a sharp drop. The peak stress, which is used as a measure of strength, varies with the metallic element of interfacial layers. Likewise, the elastic modulus, reflected by the slope of the curves in the elastic region, and the elastic strain also depend on the metallic element of interfacial layers. The strength of samples ($\sigma_{\mathrm{Strength}}$) can be evaluated by the following expression:

(1) $$\sigma_{\mathrm{Strength}}=E\varepsilon$$

where E and ε are the elastic modulus and elastic strain of the samples, respectively. These observations indicate that the introduction of interfacial layers modulates the elastic modulus and elastic strain, thereby influencing the strength of the samples. For a quantitative comparison, the variation in strength across samples with different interfacial layers is shown in Figure 2b. The samples are ranked in descending order of strength as Cu/Ni-Pd, Cu/Ni-Ag, Cu/Ni-Au, Cu/Ni-Pb, Cu/Ni-Al, and Cu/Ni-Pt, with values ranging from 8.14 GPa to 7.05 GPa. These results confirm that the strength of Cu/Ni nanolayered composites with interfacial layers is governed by the metallic elements of the interfacial layers.

**Figure 3:**
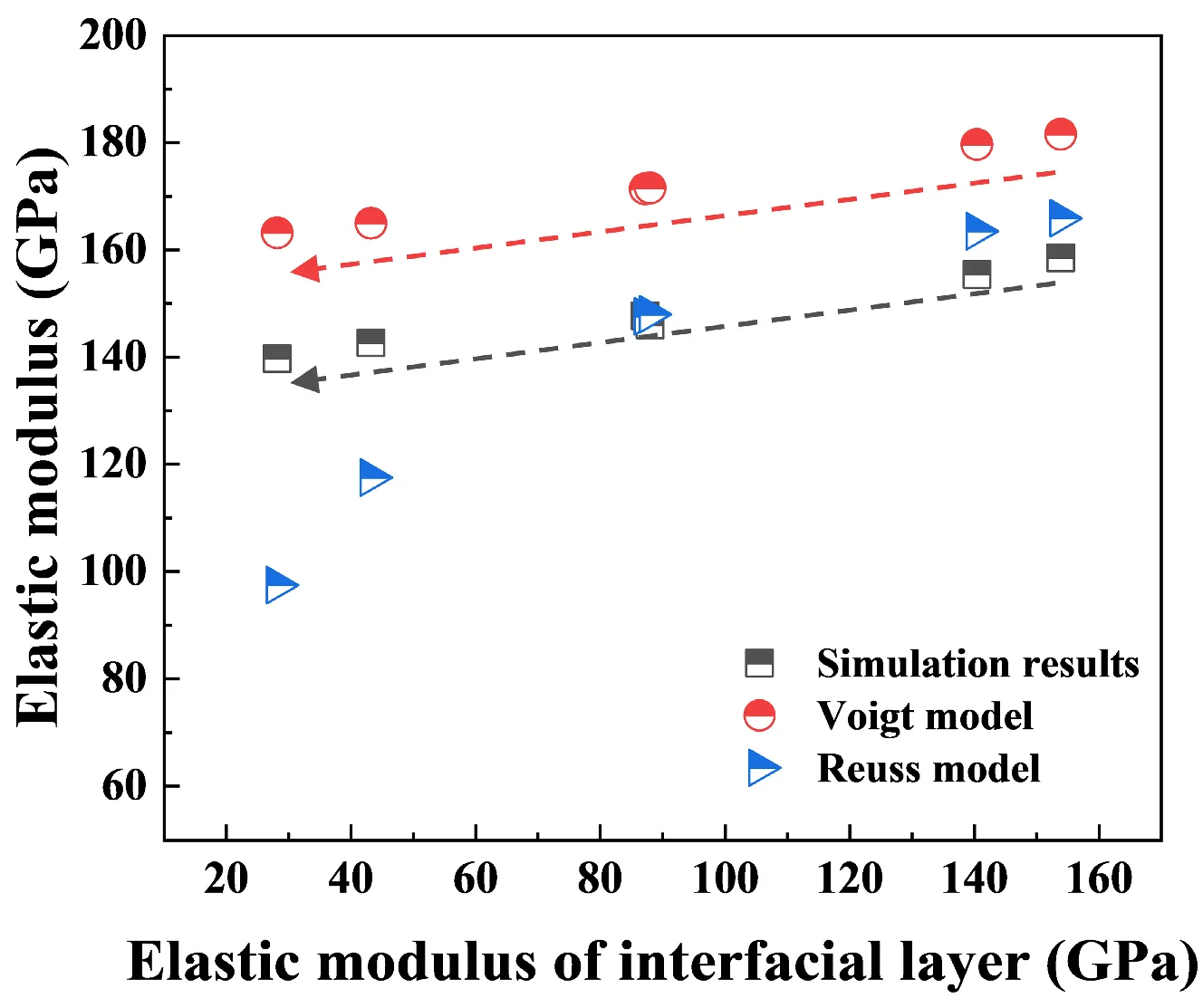
Relationship between the elastic modulus of the samples and that of interfacial layers, including simulation data and the predictions from the Voigt and Reuss models.

To explore the effect of the metallic elements of the interfacial layer on the strength of samples, the elastic modulus and elastic strain were investigated. The relationship between the elastic modulus of samples and that of interfacial layers is presented in Figure 3. Based on iso-strain and iso-stress assumptions, the elastic modulus of samples can be predicted by the Voigt and Reuss models, respectively. The predicted values of the Voigt model ($E_{V}$) are calculated as follows:

(2) $$E_{V}=\frac{E_{\mathrm{Cu}}h_{\mathrm{Cu}}}{h}+\frac{E_{1}h_{1}}{h}+\frac{E_{\mathrm{Ni}}h_{\mathrm{Ni}}}{h}+\frac{E_{2}h_{2}}{h}$$

and those of the Reuss model ($E_{R}$) are given by:

(3) $$\frac{1}{E_{R}}=\frac{h_{\mathrm{Cu}}}{E_{\mathrm{Cu}}h}+\frac{h_{1}}{E_{1}h}+\frac{h_{\mathrm{Ni}}}{E_{\mathrm{Ni}}h}+\frac{h_{2}}{E_{2}h}$$

where $E_{\mathrm{Cu}}$, $E_{\mathrm{Ni}}$, and $E_{i}$ are the elastic modulus of Cu, Ni, and interfacial layers, respectively; and $h_{\mathrm{Cu}}$, $h_{\mathrm{Ni}}$, $h_{i}$, and h are the thickness of Cu, Ni, interfacial layers, and the total samples, respectively. As shown in Figure 3, the elastic modulus of samples exhibits a linear dependence on that of the interfacial layers, which is consistent with the Voigt model predictions. This consistency suggests that the samples deform under an iso-strain condition under tension parallel to the interface. The elastic modulus of samples can be estimated from that of interfacial layers using the Voigt model.

**Figure 4:**
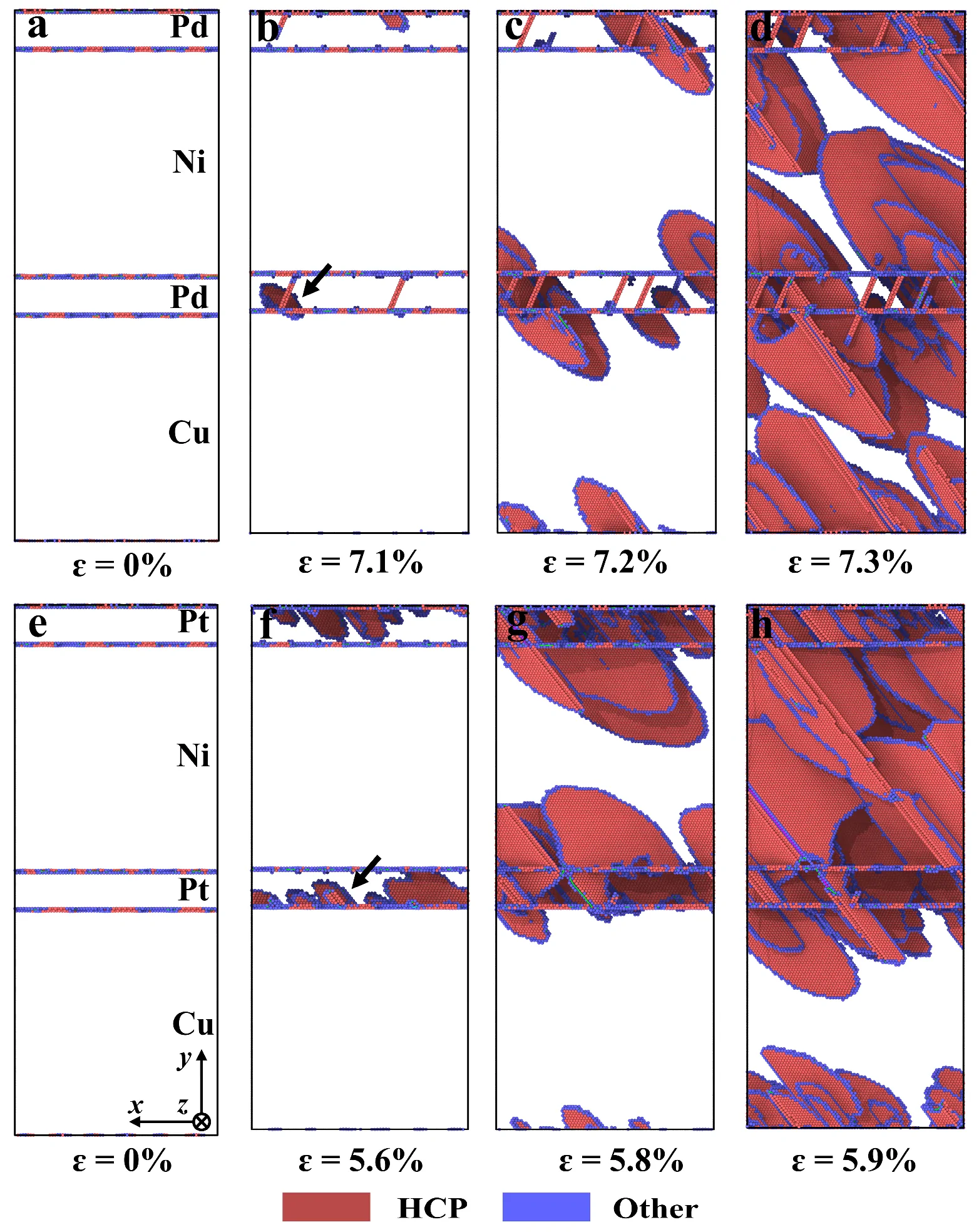
Microstructure evolution of different samples: (**a**–**d**) Cu/Ni-Pd samples and (**e**–**h**) Cu/Ni-Pt samples.

The elastic strain of samples is governed by the transition from elastic to plastic deformation, which is mediated by dislocation nucleation. The microstructure evolution of six samples was systematically investigated. Figure 4 shows the microstructure evolution of Cu/Ni-Pd and Cu/Ni-Pt samples, in which the elastic modulus of interfacial layers exceeds that of Cu layers. For clarity, atoms with a face-centered cubic (FCC) structure are deleted, while those with a hexagonal close-packed (HCP) structure are colored red, and all other atoms are shown in blue. For the Cu/Ni-Pd sample, no stacking faults are observed within each layer at initial stage (Figure 4a). At 7.1% strain, dislocation nucleation initiates at the Cu-Pd interfaces. These dislocations subsequently glide within the Pd layers, leaving behind some stacking faults (Figure 4b). After that, dislocations rapidly propagate into the Cu and Ni layers, as evidenced by the formation of numerous stacking faults in both layers (Figure 4c,d). Plastic deformation initiates via dislocation nucleation and quickly propagates to the entire sample through dislocation slip. This deformation mechanism corresponds to the stress–strain response of the Cu/Ni-Pd sample in Figure 2a: stress increases linearly with strain in the elastic region and then drops sharply upon the onset of plastic deformation. The rapid propagation of plasticity throughout the sample results in a sharp stress drop. A similar phenomenon has been observed in the Cu/Ni-Pt sample (Figure 4e–h).

**Figure 5:**
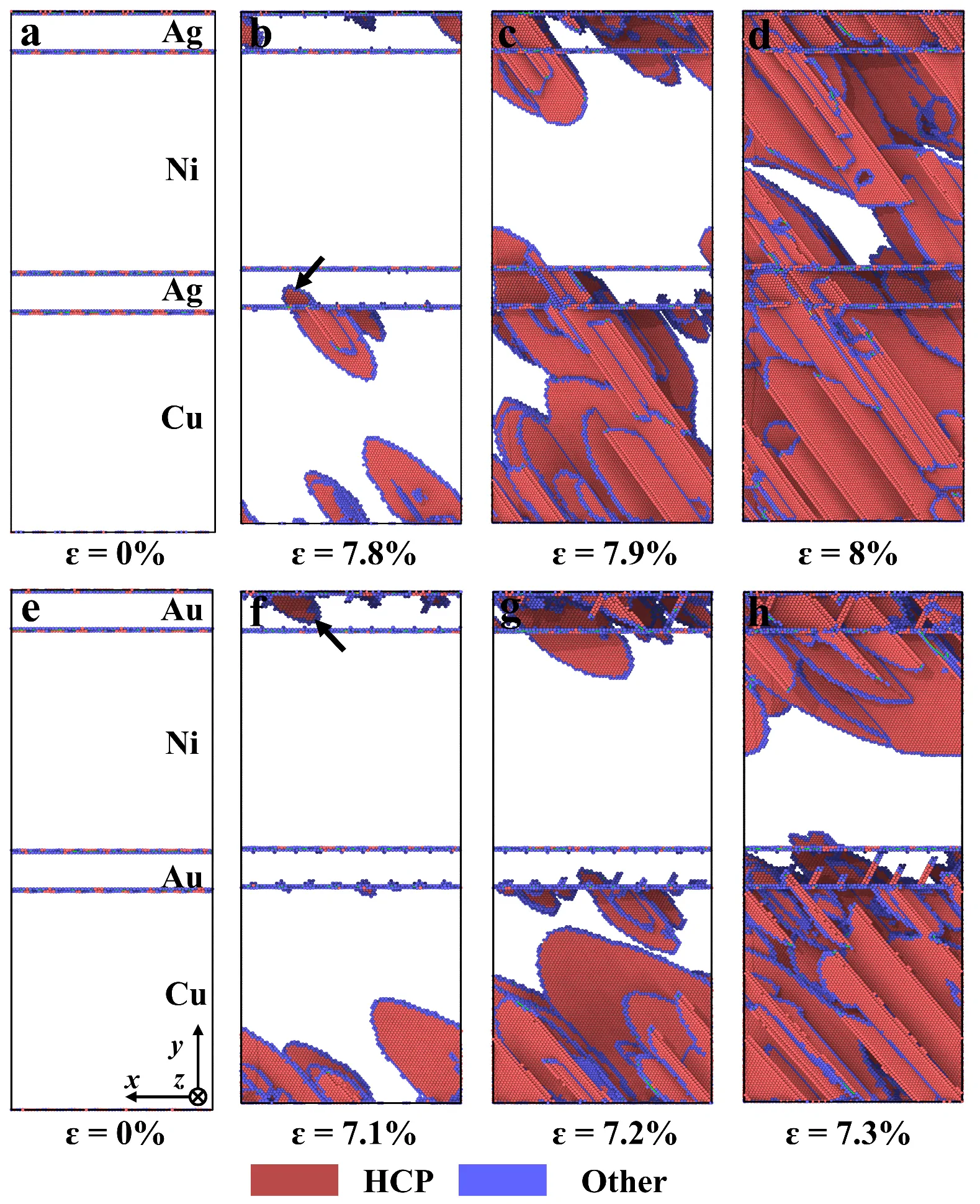
Microstructure evolution of different samples: (**a**–**d**) Cu/Ni-Ag sample and (**e**–**h**) Cu/Ni-Au samples.

In the Cu/Ni-Ag and Cu/Ni-Au samples, the elastic modulus of interfacial layers is slightly lower than that of the Cu layers. Figure 5 presents the microstructure evolution of these two samples. In the Cu/Ni-Ag sample, no stacking faults are observed before 7.8% strain (Figure 5a,b). The appearance of stacking faults in Cu and Ag layers confirms that dislocation nucleation occurs at the Cu-Ag interface and then glides into Cu and Ag layers. These dislocations glide into both Cu layers and interfacial layers simultaneously. Subsequently, dislocations propagate rapidly into the Ni layers (Figure 5c,d). The strain interval between the dislocation nucleation and the full plasticity in the sample is only 0.2%. This rapid propagation of plastic deformation corresponds to the sharp stress drop observed in Figure 2a. A similar microstructure evolution is observed for the Cu/Ni-Au sample (Figure 5e–h).

**Figure 6:**
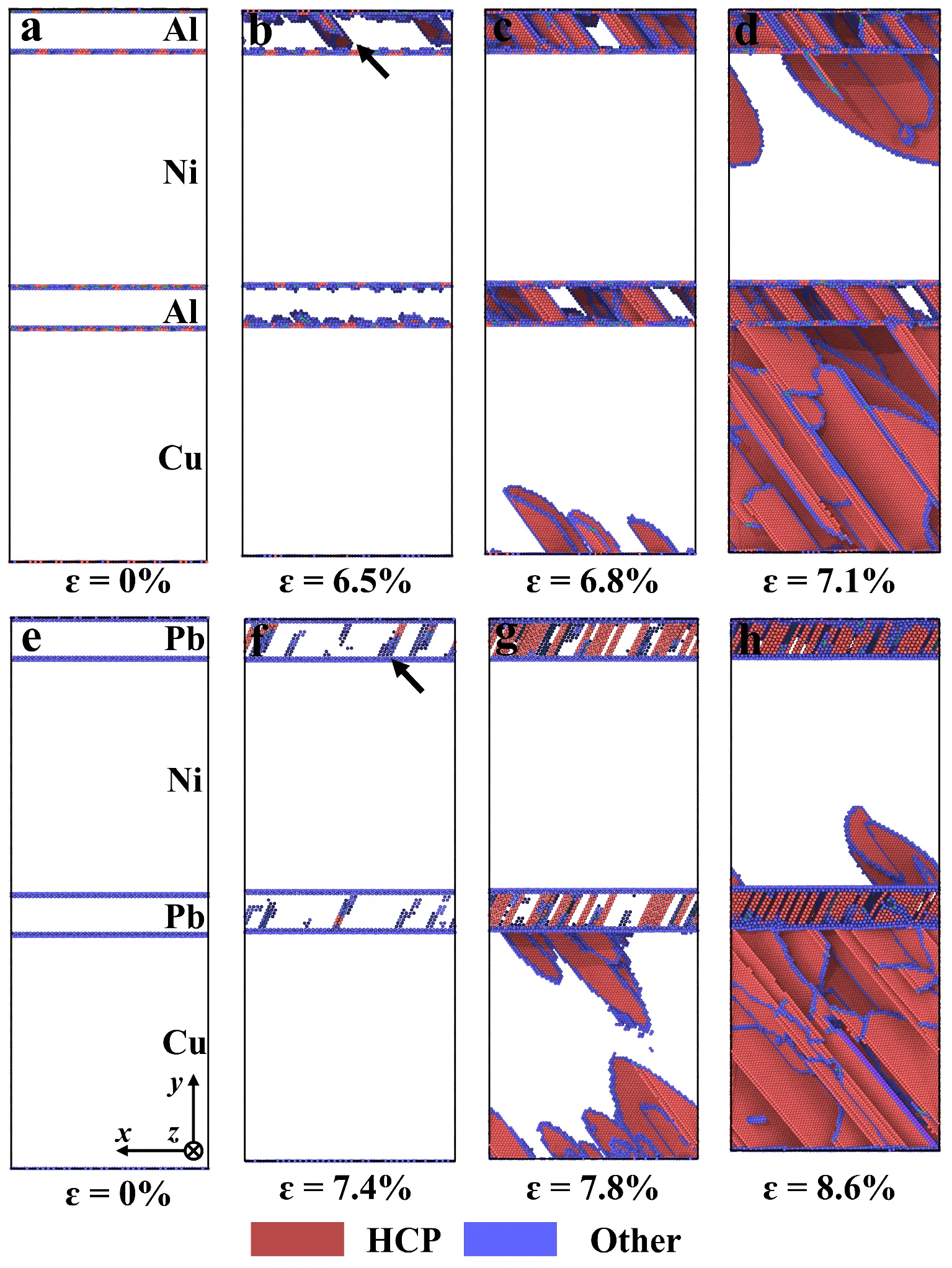
Microstructure evolution of different samples: (**a**–**d**) Cu/Ni-Al sample and (**e**–**h**) Cu/Ni-Pb samples.

For the Cu/Ni-Al and Cu/Ni-Pb samples, the elastic modulus of interfacial layers is much lower than that of the Cu layers. Figure 6 shows the microstructure evolution of these two samples. In the Cu/Ni-Al sample, dislocation nucleation is observed in the Al interfacial layers at 6.5% strain, which is confirmed by the formation of numerous stacking faults (Figure 6a,b). These dislocations are confined within the Al interfacial layers up to 6.8% strain (Figure 6c). Subsequently, the dislocations glide into the Cu layers and remain confined within both the Cu and Al layers until 7.1% strain (Figure 6d). The strain interval between the onset of deformation in different layers indicates a strong effect of interfaces on obstructing dislocation slip across layers. Similar to the Cu/Ni-Al sample, stacking faults are first observed within the interfacial layers in the Cu/Ni-Pb samples at a strain of 7.4% (Figure 6e,f). A larger strain interval between the deformation of each layer is observed (Figure 6g,h), indicating a stronger obstruction by the interfaces to dislocation.

**Figure 7:**
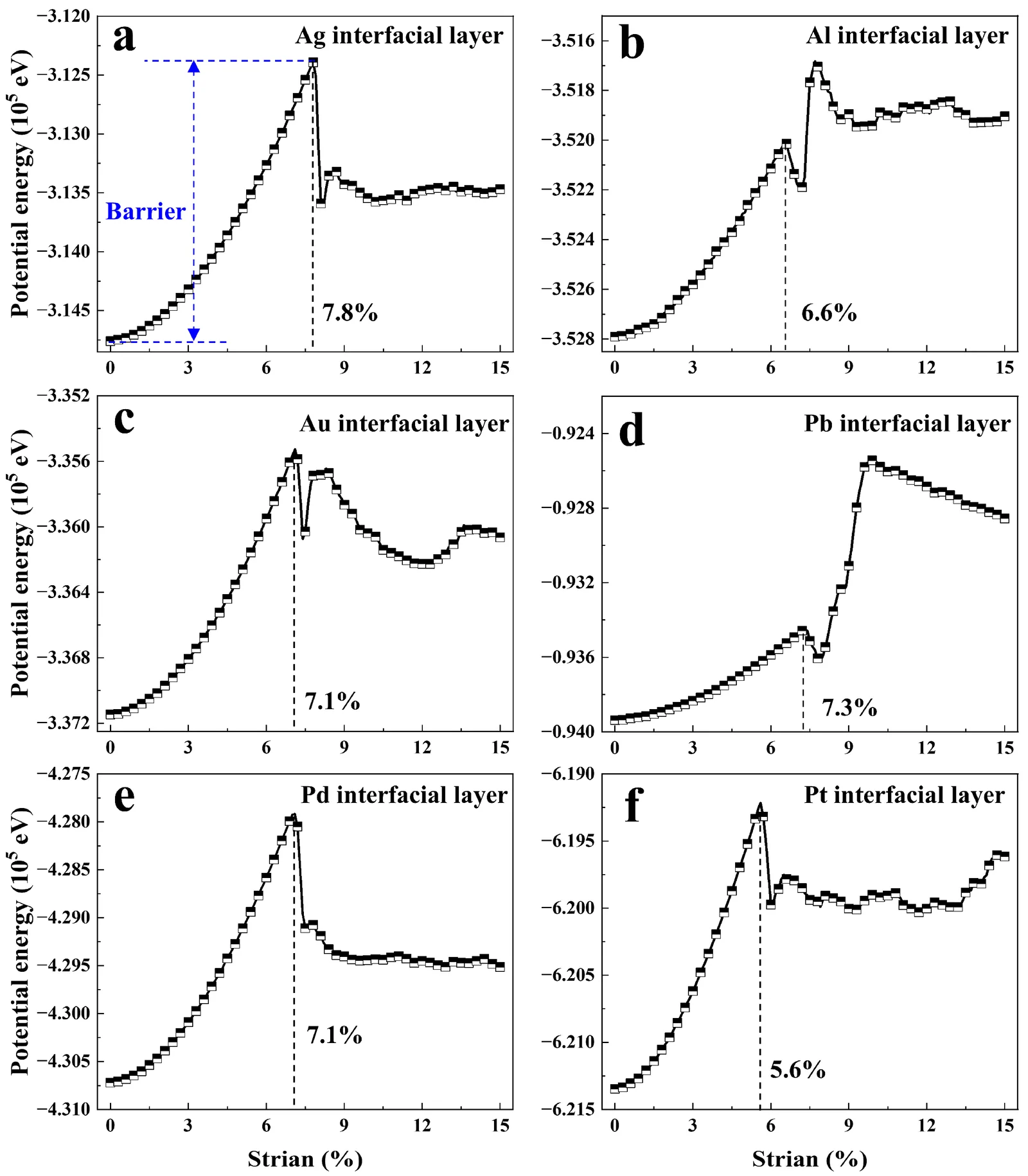
Potential energy as a function of strain for interfacial layer in different samples: (**a**) Cu/Ni-Ag; (**b**) Cu/Ni-Al; (**c**) Cu/Ni-Au; (**d**) Cu/Ni-Pb; (**e**) Cu/Ni-Pd; (**f**) Cu/Ni-Pt.

As revealed in Figure 4, Figure 5 and Figure 6, dislocation nucleation first occurs in the interfacial layer, which signals the onset of plastic deformation. The elastic strain is therefore associated with the difficulty of dislocation nucleation in interfacial layers. To quantify this difficulty, the variation in potential energy of interfacial layer with strain during the tensile simulation of samples with interfacial layers was investigated. An energy-controlled dislocation nucleation mechanism is revealed. Taking the Cu/Ni-Ag sample as an example, Figure 7a presents the variation in potential energy of Ag interfacial layers with strain. Atoms residing at interfaces (two atomic layers) were excluded from the potential energy calculation. The potential energy of the Ag interfacial layer increases as strain increases, reaches a peak value at 7.8% strain, and then decreases. Importantly, the strain at which the energy drop initiates in the Ag interfacial layers coincides with the strain at which the stress drop occurs. These observations indicate that dislocation nucleation is triggered once the accumulated energy reaches a critical threshold. Consequently, the energy barrier of the interfacial layer where dislocation nucleation first occurs can serve as a quantitative indicator of the difficulty of dislocation nucleation. Similar barriers are observed for other interfacial layers (Figure 7b–f).

**Figure 8:**
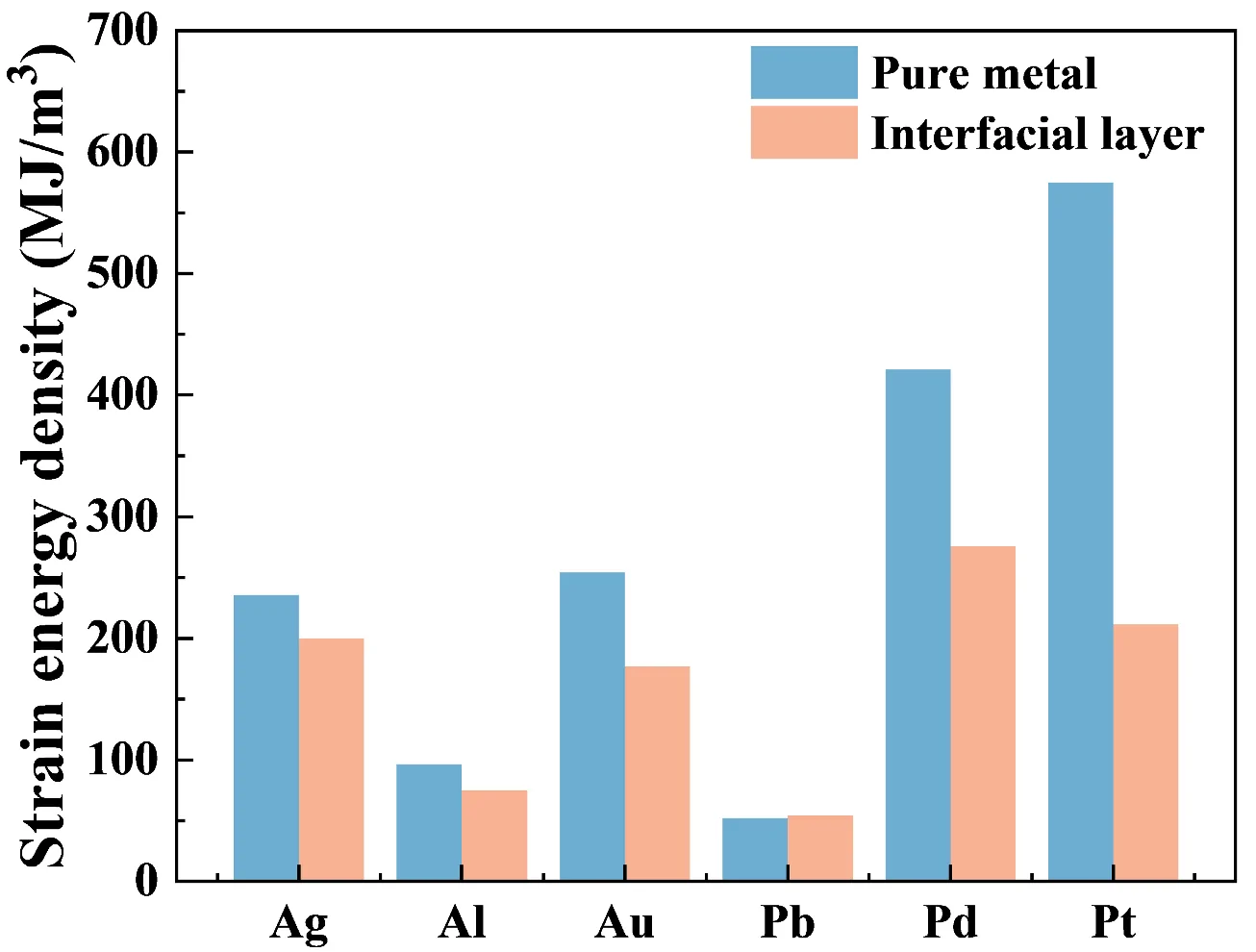
Variation in strain energy density required for dislocation nucleation with different interfacial layers, the corresponding results for their pure metals.

For a quantitative comparison of the energy barriers required for dislocation nucleation across different interfacial layers, the strain energy density, defined as the energy barrier divided by the volume of interfacial layers, is calculated. The volume of interfacial layers does not include the volume of atoms at the interface. The strain energy density of the corresponding pure metal is also included. The simulation condition of pure metals is the same as that used in the simulation of samples with interfacial layers. As shown in Figure 8, pure Pt exhibits the highest strain energy density of 574.9 MJ/m^3^, followed by Pd, Au, Ag, Al, and Pb in descending order. Pure Pb exhibits a significantly lower strain energy density required for dislocation nucleation than other metals. This phenomenon is related to the weak metallic bonding of Pb, which indicates a low energy barrier for dislocation motions [49,50]. However, the strain energy density of interfacial layers is much lower than that of their pure metal counterparts. For instance, the Pt interfacial layer shows a strain energy density of 211.5 MJ/m^3^. These results indicate that dislocation nucleation in pure metals with no defect requires a high strain energy density threshold, whereas the presence of interfaces leads to a sharp reduction in strain energy density threshold. Notably, the extent of this reduction varies significantly across different interfacial layers. The reduction from pure Pt to Pt interfacial layers is as large as 363.4 MJ/m^3^, whereas that from pure Al to Al interfacial layers is only 21.3 MJ/m^3^, demonstrating a strong dependence on the metallic element of interfacial layers.

**Figure 9:**
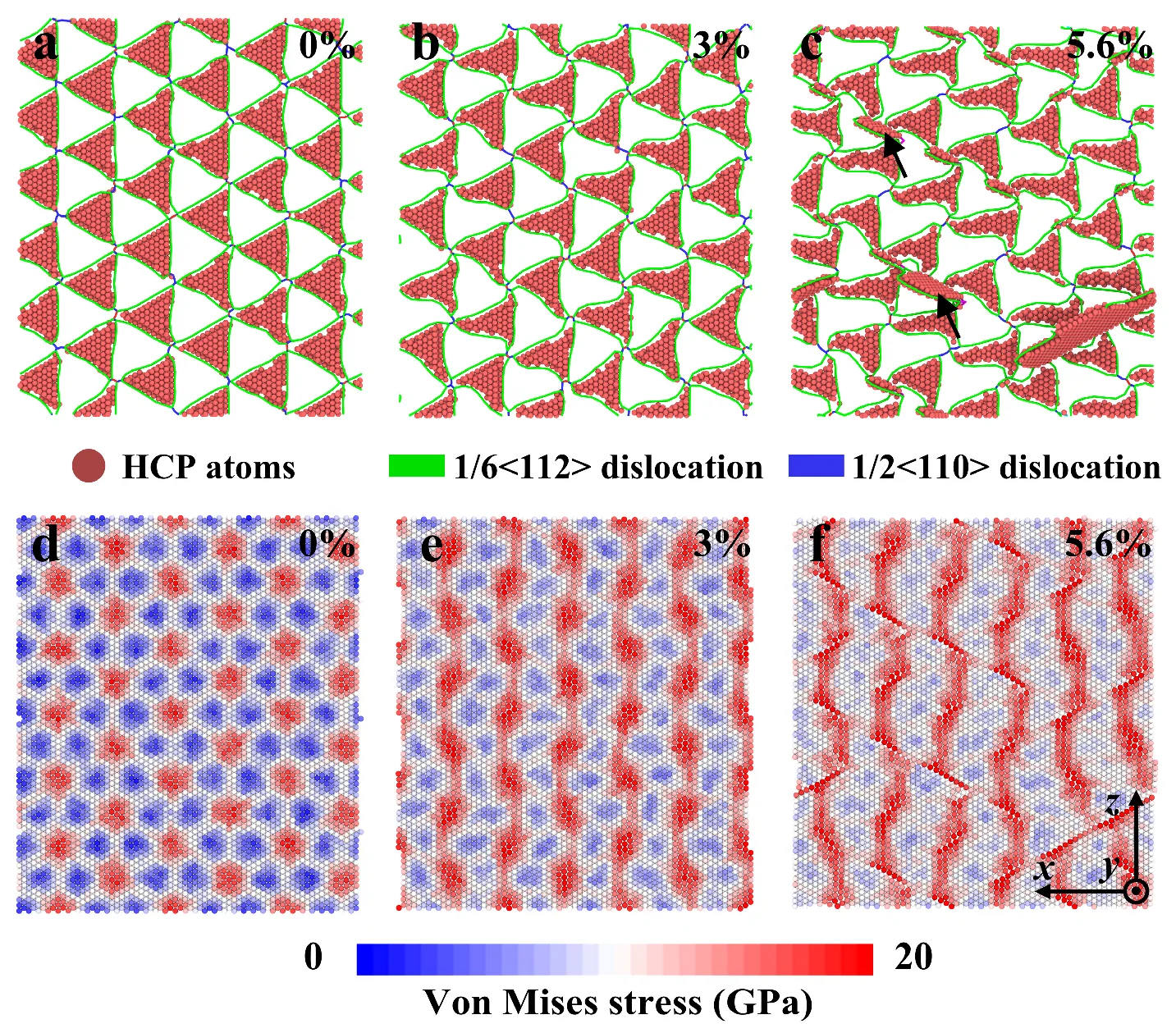
Evolution of microstructures and von Mises stress distribution in the Cu/Ni-Pt samples. (**a**–**c**) Microstructure evolution of the Cu-Pt interfaces under different strains; (**d**–**f**) the corresponding von Mises stress distribution. The arrow indicates dislocation nucleation at node.

To explore the underlying mechanism of the varying reduction in strain energy density required for dislocation nucleation across different interfacial layers, the stress distribution at the interfaces is investigated. The atomic von Mises stress is calculated as follows:

(4) $$\sigma_{\mathrm{von}}=\sqrt{\frac{{\left(\sigma_{xx}-\sigma_{yy}\right)}^{2}+{\left(\sigma_{xx}-\sigma_{zz}\right)}^{2}+{\left(\sigma_{yy}-\sigma_{zz}\right)}^{2}+6(\tau_{xy}^{2}+\tau_{yz}^{2}+\tau_{zx}^{2})}{2}}$$

where $\sigma_{xx}$, $\sigma_{yy}$, $\sigma_{zz}$, $\tau_{xy}$, $\tau_{xz}$, and $\tau_{yz}$ are the stress components. The microstructural evolution and the corresponding von Mises stress distribution of interfaces were presented in Figure 9. Taking the Cu/Ni-Pt sample as an example, a dislocation network forms at the initial stage due to the lattice mismatch (Figure 9a). The stress distribution reveals that stress initially concentrates along dislocation lines, particularly at the network nodes (Figure 9d). As strain increases to 3%, the dislocation lines become curved, and the stress concentration intensifies (Figure 9b,e). At 5.6% strain, dislocation nucleation occurs at the network nodes, as indicated by the arrow in Figure 9c. These results demonstrate that dislocations preferentially nucleate from the nodes of the dislocation network, where stress concentration is most severe.

**Figure 10:**
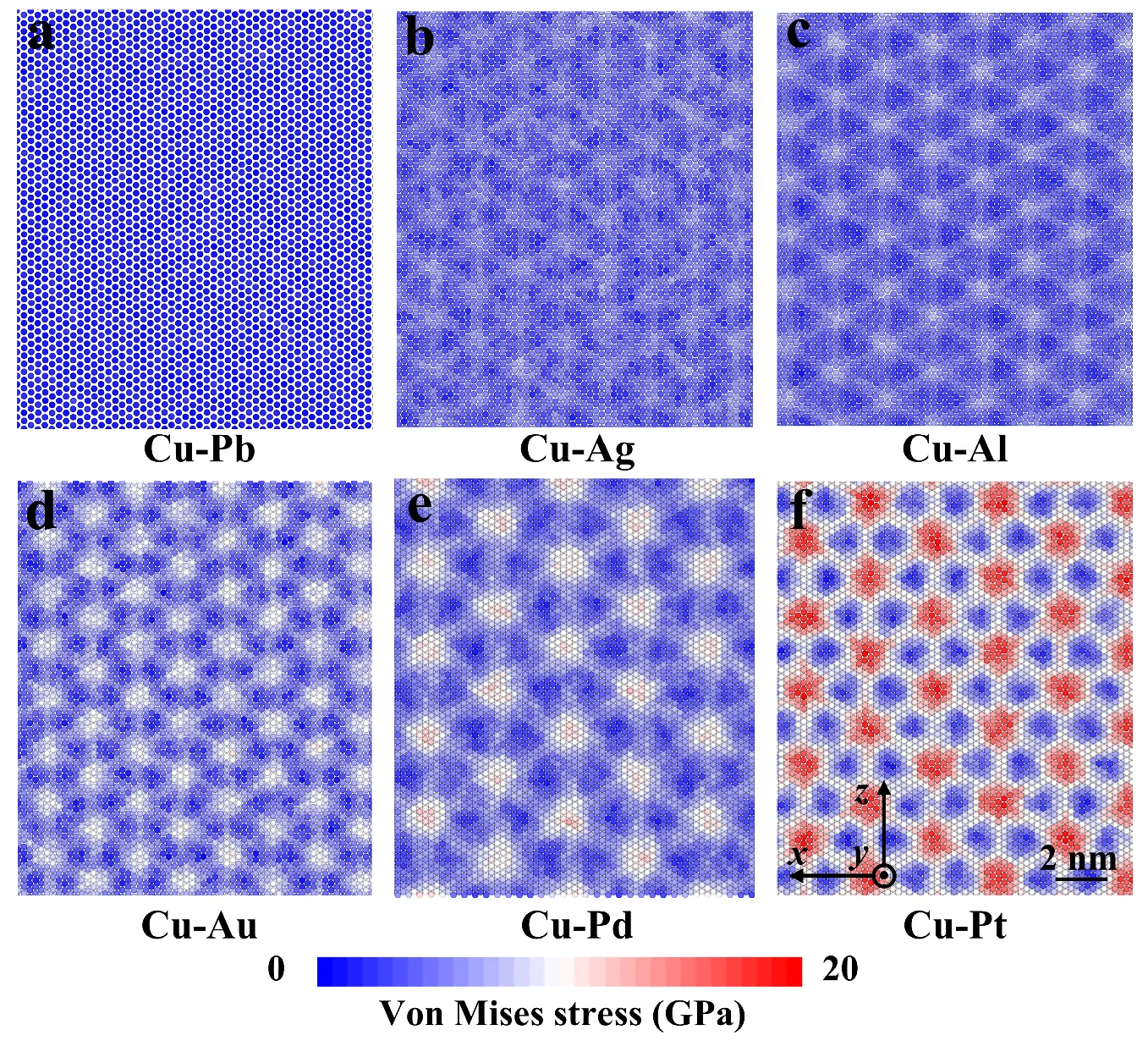
Distribution of von Mises stress in different interfaces: (**a**) Cu-Pb; (**b**) Cu-Ag; (**c**) Cu-Al; (**d**) Cu-Au; (**e**) Cu-Pd; (**f**) Cu-Pt.

Figure 10 shows the stress distribution of the six interfaces at the initial stage. For the Cu-Pb interfaces in the Cu/Ni-Pb sample, no significant stress concentration is observed (Figure 10a). In the Cu/Ni-Ag sample, a slight stress concentration is observed, as confirmed by the appearance of a white region with higher stress than the surrounding areas (Figure 10b). The stress concentration becomes progressively more pronounced as the interfaces change from Cu-Ag, Cu-Al, Cu-Au, Cu-Pd, and Cu-Pt. In the Cu-Pt interfaces, atomic stresses as high as 20 GPa are detected, indicating a severe stress concentration. Such a stress concentration promotes dislocation nucleation, leading to the reduction in the strain energy density threshold. Note that the observed variation in the threshold between the interfacial layers and pure metals is primarily attributed to the introduction of defects rather than the thickness of interfacial layers (Supplementary Figure S1).

**Figure 11:**
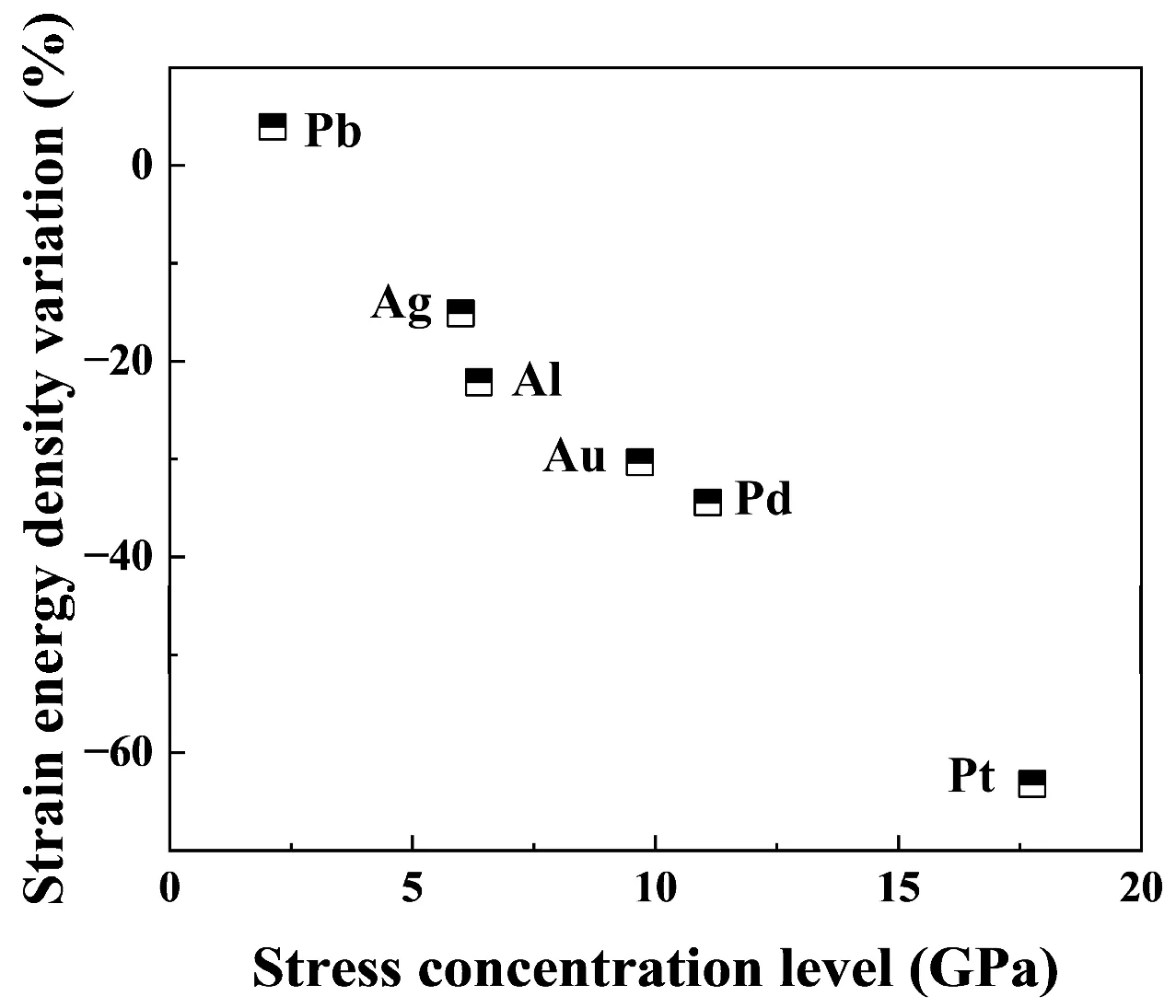
Strain energy density variation between pure metal and interfacial layers varies with the stress concentration level of interfaces.

Furthermore, the relationship between the strain energy density variation and stress concentration level was investigated. To quantify the stress concentration level, atoms with von Mises stress in the top 5% were selected, and their average stress was calculated as the stress concentration level. As shown in Figure 11, the strain energy density variation exhibits a linear dependence on the stress concentration level: a higher stress concentration corresponds to a greater reduction in strain energy density. The strain energy density required for dislocation nucleation in interfacial layers ($W_{\mathrm{IL}}$) can be presented as follows:

(5) $$W_{\mathrm{IL}}=W_{P}\lambda_{\mathrm{IL}}$$

where $W_{P}$ is the strain energy density required for dislocation nucleation in pure metal, and $\lambda_{\mathrm{IL}}$ is a parameter related to the stress concentration level at interfaces. In addition, the strain energy density threshold of the interfacial layer can also be calculated as follows:

(6) $$W_{\mathrm{IL}}=\frac{E_{\mathrm{IL}}\varepsilon_{\mathrm{IL}}^{2}}{2}$$

where $E_{\mathrm{IL}}$ is the elastic modulus of interfacial layers and $\varepsilon_{\mathrm{IL}}$ is the elastic strain of interfacial layers as well as the elastic strain of samples due to the iso-strain condition. Note that Equation (6) is valid only when the stress–strain curves exhibit a strictly linear elastic response. Under this condition, the curve forms a right triangle with the strain axis, and the strain energy density is simply the area of this triangle. However, the stress–strain curve in the present study does not exhibit a strict right-angled triangular shape, as the slope of curves reduces with increasing strain (Figure 2a). Consequently, the strain energy density calculated by Equation (6) is overestimated relative to the actual value, necessitating a correction parameter. The formula is thus revised as:

(7) $$W_{\mathrm{IL}}=\alpha \frac{E_{\mathrm{IL}}\varepsilon_{\mathrm{IL}}^{2}}{2}$$

where α is the correction parameter. The elastic strain of composites can be calculated as follows:

(8) $$\varepsilon =\sqrt{\frac{2W_{P}\lambda_{\mathrm{IL}}}{\alpha E_{\mathrm{IL}}}}$$

and the strength of composites can be evaluated as:

(9) $$\sigma_{\mathrm{Strength}}=(\frac{E_{\mathrm{Cu}}h_{\mathrm{Cu}}+2E_{\mathrm{IL}}h_{\mathrm{IL}}+E_{\mathrm{Ni}}h_{\mathrm{Ni}}}{h})\sqrt{\frac{2W_{P}\lambda_{\mathrm{IL}}}{\alpha E_{\mathrm{IL}}}}$$

Overall, these results indicate that selecting metals with a high elastic modulus, high strain energy density required for dislocation nucleation, while simultaneously designing interfaces with low stress concentration, can effectively enhance the strength of nanolayered composites with interfacial layers.

## 4. Conclusions

In this study, the tensile properties of Cu/Ni nanolayered composites with Ag, Al, Au, Pb, Pd, and Pt interfacial layers under loading parallel to the interfaces were systematically investigated via molecular dynamics simulations. The main conclusions are summarized as follows.

Our simulation results show that the strength of Cu/Ni composites with interfacial layers is dependent on the metallic element of interfacial layer. Composites with Pd interfacial layers possess the highest strength, followed sequentially by composites with Ag, Au, Pb, Al and Pt interfacial layers, with strength values ranging from 8.14 GPa to 7.05 GPa. The strength of composites can be evaluated by the product of the elastic modulus and the elastic strain. The elastic modulus of composites scales linearly with that of interfacial layers, in good agreement with the Voigt model, indicating that the composites deform under an iso-strain condition when loaded parallel to the interfaces. Based on dislocation nucleation behavior, we established the relationship between the elastic strain of composites and properties of pure metals used for interfacial layers, such as strain energy density required for dislocation nucleation, stress concentration level and elastic modulus. The strength of composites with interfacial layers under tension parallel to the interfaces arises from the synergistic interplay of the above three characteristics of interfacial layers. Selecting metals with high strain energy density required for dislocation nucleation and high elastic modulus and constructing interfaces with low stress concentration can enhance the strength of composites with interfacial layers.

The present study elucidates the key parameter governing the strength of Cu/Ni nanolayered composites with interfacial layers and establishes a quantitative framework linking the strength of composites to the intrinsic properties of the metals used for interfacial layers. These findings provide a fundamental basis for the design of high-performance nanolayered metallic composites.

## Data Availability

The original contributions presented in this study are included in the article. Further inquiries can be directed to the corresponding author.

## Supplementary Materials

The following supporting information can be downloaded at: https://www.mdpi.com/article/10.3390/nano16171106/s1, Figure S1: Simulation results for Cu/Nb nanolayered composites with Ag interfacial layers of 7 nm thickness. (a) Atomic configuration of the simulation sample. (b) Potential energy as a function of strain for the simulation sample. (c) Strain energy density required for dislocation nucleation for samples with Ag interfacial layers of different thickness.
