# Supplementary material for: A Predictive Strength Model for Cu/Ni Nanolayered Composites with FCC Interfacial Layers Under Loading Parallel to the Interface
Source: Nanomaterials (Basel). 2026 Sep 2;16(17):1106. doi: 10.3390/nano16171106 (PMC13567483; doi:10.3390/nano16171106)
Supplement: Supplementary file 1 [file nanomaterials-16-01106-s001.zip › nanomaterials-4535512-supplementary.pdf]

Supplementary Materials to "A predictive strength model for Cu/Ni nanolayered composites with FCC interfacial layers under loading parallel to the interface"

Yaodong Wang[a,*], Jianjun Li[b]

[a] *State Key Laboratory of Intelligent Construction and Healthy Operation and Maintenance of Deep Underground Engineering, School of Mechanics and Civil Engineering, China University of Mining and Technology, Xuzhou, Jiangsu, 221116, China*

[b] *School of Mechanics and Engineering Science, Ningbo University, Ningbo, 315211, China*

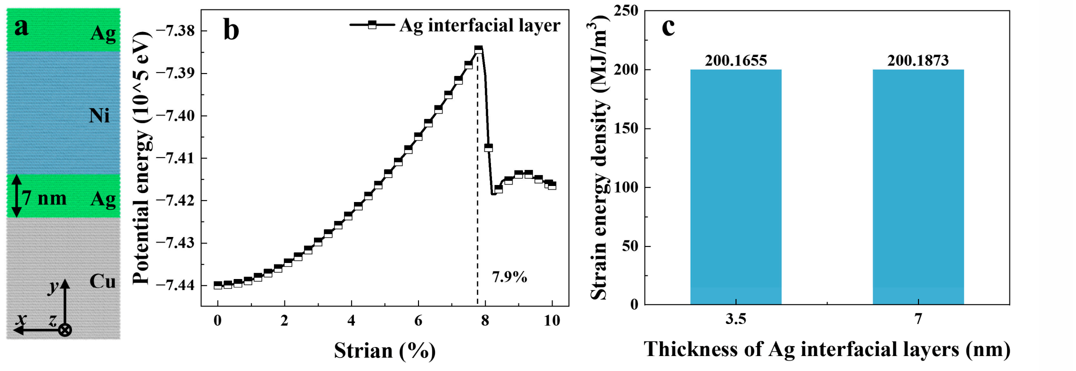

Figure S1. Simulation results for Cu/Nb nanolayered composites with Ag interfacial layers of 7 nm thickness. (a) Atomic configuration of the simulation sample. (b) Potential energy as a function of strain for the simulation sample. (c) Strain energy density required for dislocation nucleation for samples with Ag interfacial layers of different thickness.

* Corresponding author. E-mail addresses: wangyd@cumt.edu.cn.
